# Ultrarapid Endoscopic-Aided Hematoma Evacuation in Patients with Thalamic Hemorrhage

**DOI:** 10.1155/2021/8886004

**Published:** 2021-01-19

**Authors:** Kuan-Yu Chen, Woon-Man Kung, Lu-Ting Kuo, Abel Po-Hao Huang

**Affiliations:** ^1^School of Medicine, National Taiwan University, Taipei, Taiwan; ^2^Department of Exercise and Health Promotion, College of Kinesiology and Health, Chinese Culture University, Taipei, Taiwan; ^3^Division of Neurosurgery, Department of Surgery, National Taiwan University Hospital and National Taiwan University College of Medicine, Taipei, Taiwan

## Abstract

Thalamic hemorrhage bears the worst outcome among supratentorial intracerebral hemorrhage (ICH). Minimally invasive endoscopic-aided surgery (MIS) has been proved to be safe and effective in evacuating ICH. However, the ideal timing of MIS is still a controversy. In this study, we present our experience in the treatment of patients with thalamic hemorrhage by ultrarapid MIS evacuation. This retrospective analysis enrolled seven patients treated with ultrarapid MIS evacuation of thalamic hemorrhage. Seven patients treated with EVD with similar ICH score were included as match control. Primary endpoints included rebleeding, morbidity, and mortality. Hematoma evacuation rate was evaluated by comparing the pre- and postoperative computed tomography (CT) scans. Glasgow Outcome Scale Extended (GOSE) and modified Rankin Score (mRS) were noted at the 6-month and 1-year postoperative follow-up. Among the seven patients, six were accompanied with intraventricular hemorrhage. All patients received surgery within 6 hours after the onset of stroke. The mean hematoma volume was 35 mL, and the mean operative time was 116.4 minutes. The median hematoma evacuation rate was 74.9%. There was no rebleeding or death reported after the surgery. The median GOSE and mRS were 3 and 5, respectively, at 6 months postoperatively. Further, 1-year postoperative median GOSE and mRS were 3 and 5, respectively. The data suggest that the ultrarapid MIS technique is a safe and effective way in the management of selected cases with thalamic hemorrhage, with favorable long-term functional outcomes. However, a large, prospective, randomized-controlled trial is needed to confirm these findings.

## 1. Introduction

Spontaneous intracerebral hemorrhage (ICH) is a common neurosurgical emergency. The incidence of ICH is around 24 per 100,000 person-years in white people, 23 per 100,000 person-years in black people, and 52 per 100,000 person-years in Asian people [1]. Roughly 10 to 15% of ICH cases involve the thalamus. Thalamic hemorrhage bears the worst outcome among supratentorial ICH. Hematoma of the thalamus may expand and affect different proximal structures (e.g., the ventricle, globus pallidus, and internal capsule), leading to different extent of disability and mortality.

Due to its deeply seated anatomy, thalamic hemorrhage is hard to evacuate. Recently, minimally invasive endoscopic-aided surgery (MIS) for evacuating ICH has been considered as a safe and effective approach, showing with lower morbidity and mortality than the traditional craniotomy [2–4]. Many factors such as hematoma volume, intraventricular extension, and location of hematoma have been found to be associated with the prognosis of MIS [5]. However, the ideal timing to perform minimally invasive surgery (MIS) is still a controversy. It has been reported that early surgery performed within 6 to 24 hours ICH is ideal for MIS [6]; however, whether the ultrarapid MIS performed within 6 hours after ICH is favorable to patients is still under debate.

In this study, we focus on the surgical management of thalamic hemorrhage and present our experience in treating thalamic hemorrhage using ultrarapid MIS evacuation by comparing the postoperative outcomes between patients receiving ultrarapid MIS evacuation and patients using extraventricular drainage (EVD) without thalamic hematoma evacuation.

## 2. Materials and Methods

### 2.1. Patient Selection

In this study, we included patients with ICH fulfilling the following criteria: (1) thalamic hemorrhage with >20 mL hematoma volume, accompanied with or without intraventricular hemorrhage (IVH) and acute hydrocephalus and (2) had undergone MIS within 24 hours after the onset of stroke. Moreover, we included another matched (control) group with thalamic hemorrhage fulfilling the following criteria: (1) ICH score ≥ 2, (2) had undergone unilateral or bilateral EVD without thalamic hematoma evacuation, and (3) had undergone EVD placement within 24 hours after the onset of stroke. Patients were excluded if they had met anyone of the following criteria: (1) ICH caused by the trauma, tumor, and coagulopathy (prothrombin time international normalized ratio (PT INR) > 1.3, partial thromboplastin time (PTT) > 35.5 seconds, and platelet count < 100, 000/mL); (2) with end-stage renal disease or Child-Pugh Class C cirrhosis; (3) taking antiplatelet or anticoagulation medications, (4) with preoperative Glasgow coma scale (GCS) score of <4 or >14; and (5) without the data on follow-up computed tomography (CT) result within 3 days or lost to follow-up at 6 months. Both MIS and EVD groups were managed by the same clinical surgical team at National Taiwan University Hospital.

The study was designed and conducted in accordance with the applicable local regulations and the ethical principles of the Declaration of Helsinki and was approved by Institutional Review Board (IRB) of National Taiwan University Hospital (IRB number: 201611058RINA). Written informed consent was waived as this is a retrospective research.

### 2.2. For the Removal of IVH

We used the ipsilateral Kocher point as the entry point for MIS and inserted external ventricular drain (EVD) through the operative tract for removing the IVH. A flexible endoscope with the free-hand technique was applied to evacuate massive hematoma in the third and fourth ventricles of patients with massive IVH. Also, bilateral EVD placement might be conducted.

Under general anesthesia, a 3.5 to 4.0 cm linear skin incision was made, followed by a burr hole (1.5-2.0 cm in diameter) drilling. Intraoperative sonography (ALOKA Prosound alpha 5 SV with UST-5268P-5 Multi-Frequency Phased Array Burr-hole Probe, 3.0-7.5 MHz, Tokyo, Japan) was applied to locate hematoma or ventricle before conducting cruciate durotomy and small corticotomy. A custom-made transparent plastic sheath (10 mm in outer diameter, with various lengths depending on surgeon's choice as well as the estimated length measured on preoperative CT scan) was inserted along the planned trajectory with the stylet. After removal of the stylet, the endoscope (4 mm, 0° rod-lens endoscope, and 18 cm in length; Karl Storz, Tuttlingen, Germany) was introduced into the transparent sheath to provide visualization when removing the hematoma.

Following our procedure of hematoma removal described in previous studies [4, 7], we entered the ventricle to evacuate IVH first. Next, we identified the rupture site of thalamic hemorrhage and evacuated the hematoma using the penetrating technique with an 8 Fr. angled suction in the working space of the sheath. A flexible endoscope (outer diameter: 2.5 mm; Karl Storz) could be introduced as an alternative to facilitate the removal of hematoma and to avoid excessive rotation or significant manipulation of the sheath enclosed in the brain parenchyma.

For most cases, ICH could be evacuated without active or pronounced bleeding during the operation. For patients with intraoperative bleeding, we applied the “wait-and-see saline irrigation” method to stop the bleeding when bleeding from a small artery or perforating vessel was found. For patients with intraoperative bleeding, we applied the “wait-and-see saline irrigation” method to stop the bleeding when bleeding from a small artery or perforating vessel was found. If the bleeding had not been stopped, the balanced irrigation-suction technique was then used to identify the bleeding site [8], where we injected FloSeal Hemostatic Matrix via a specialized 3 mm flexible catheter into the hematoma cavity to achieve hemostasis. Then, cotton was used for coverage, followed by normal saline to remove the residual FloSeal Hemostatic Matrix.

### 2.3. Clinical Follow-Up

All patients were followed up by brain CT within 3 days after the operation. The hematoma evacuation rate was calculated using the following formula: [(Preoperative hematoma volume − Postoperative hematoma volume)/preoperative hematoma volume] × 100%.

Primary endpoints included rebleeding, morbidity, and mortality after the surgery. The Glasgow Outcome Scale Extended (GOSE) score and the modified Rankin Score (mRS) were evaluated at 6-month and 1-year postoperative follow-up either at an outpatient department or by phone.

### 2.4. Statistical Analysis

Descriptive analysis and linear regression were conducted using SPSS (Statistical Package for the Social Sciences). Continuous variables were presented as mean, standard deviation (SD), median, and range, while the categorical variables were summarized as number and percentage. No imputation was applied for missing data.

## 3. Results

### 3.1. Baseline Characteristics

Table 1 summarizes patients' characteristics and surgical outcome. In the MIS group, seven patients receiving MIS within 24 hours of ictus were enrolled, including four men and three women at a mean age (mean ± SD) of 66.6 ± 10.5 years (range: 53-87 years); while in the EVD group, seven patients without hematoma evacuation were enrolled, including five men and two women at a mean age (mean ± SD) of 68.2 ± 12.4 years (range: 56-81 years). In the MIS group, six out of seven patients accompanied with IVH (three men and three women), while in the EVD group, all patients had IVH. Patients in the MIS group had higher preoperative hematoma volume and ICH score than those in the EVD group; however, the MIS group demonstrated shorter hospital stay and ICU stay than the EVD group.

### 3.2. Postoperative Outcomes

Postoperative outcomes in terms of rebleeding, morbidity, death, and functional outcomes are also summarized in Table 1. The incidences of postoperative rebleeding and morbidity were both higher in the EVD group than the MIN group. In the MIN group, there was neither rebleeding nor death reported after the surgery and only one patient received ventriculoperitoneal shunt (VP shunt) after the surgery. In the EVD group, one patient had rebleeding after the surgery and four patients experienced morbidity (received VP shunt and tracheostomy). No death was reported after the surgery in both groups.

**Figure 1:**
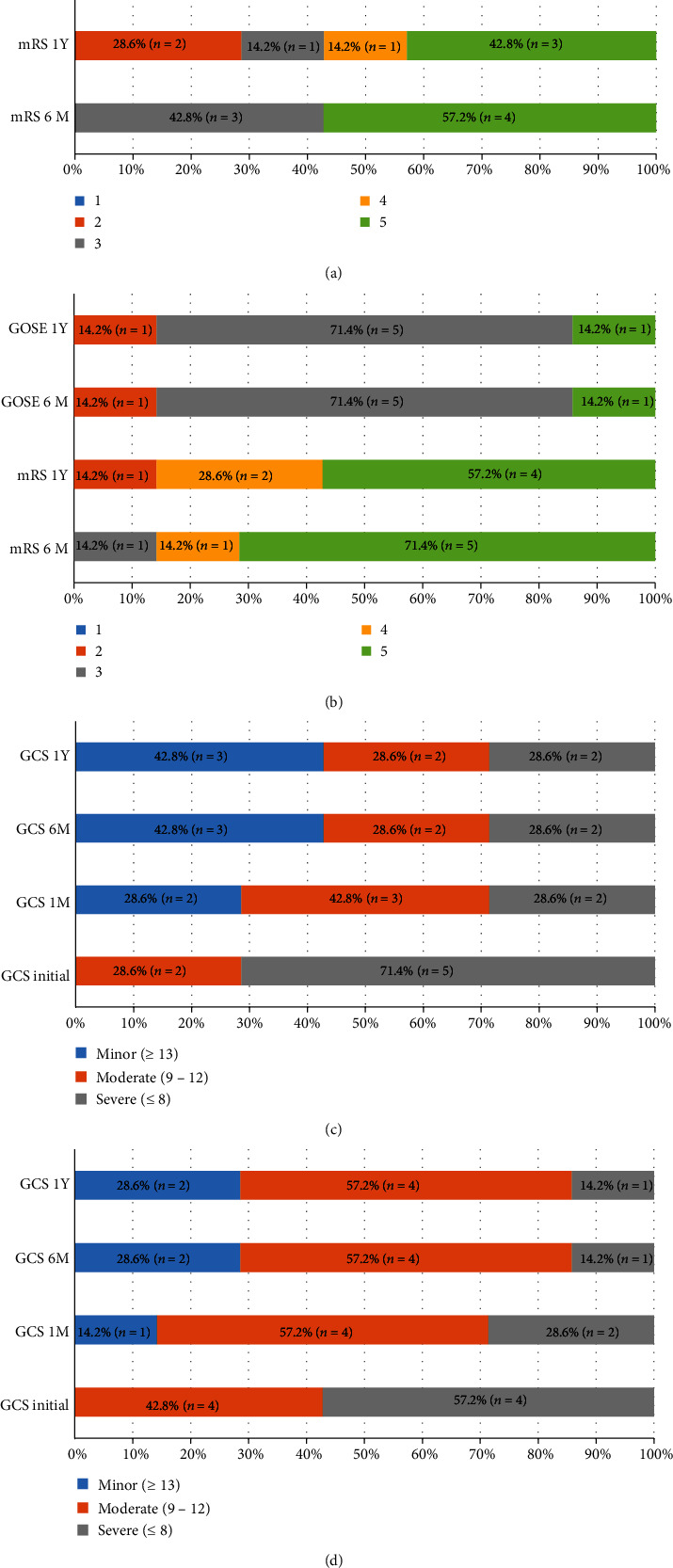
Distribution of functional outcomes. (a) Functional outcomes of the MIS group. (b) Functional outcomes of the EVD group. (c) Glasgow Coma Scale score of the MIS group. (d) Glasgow Coma Scale score of the EVD group.

For the consciousness evaluation, both groups showed improved GCS score from 8 preoperatively to 10 (MIS group) and 12 (EVD group) at 6 months postoperatively, which was sustained until 1 year. The distribution of functional outcome is presented in Figure 1, showing a sustained functional outcome in both groups after 6 months. In the MIS group, the median GOSE scores at 6 months and 1 year were both 3, with five patients graded as 3 or above. The median mRS at 1 year postoperatively was 4, with three patients showing good functional recovery (i.e., mRS ≤ 3). The EVD group demonstrated similar functional outcome as the MIS group, with a median GOSE score of 3 at both 6 months and 1 year, with six patients graded as 3 or above. The median mRS at 1 year postoperatively was 5, with one patient showing good functional recovery (i.e., mRS ≤ 3).

### 3.3. Correlation Analysis

**Figure 2:**
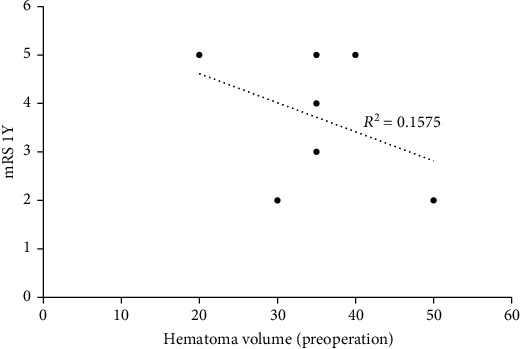
Correlation analysis of 1-year mRS and preoperative hematoma volume.

**Figure 3:**
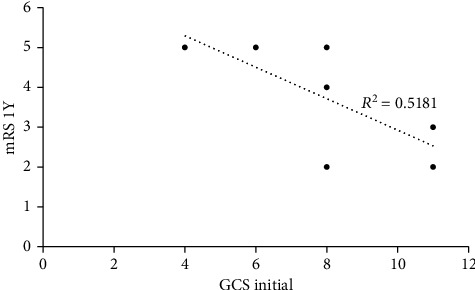
Correlation analysis of 1-year mRS and initial GCS score.

The correlation analysis of 1-year mRS and preoperative hematoma volume revealed an *R*-squared of 0.1575 (Figure 2). The correlation analysis of 1-year mRS and initial GCS score showed an *R*-squared of 0.5181 (Figure 3).

### 3.4. Illustrative Case

**Figure 4:**
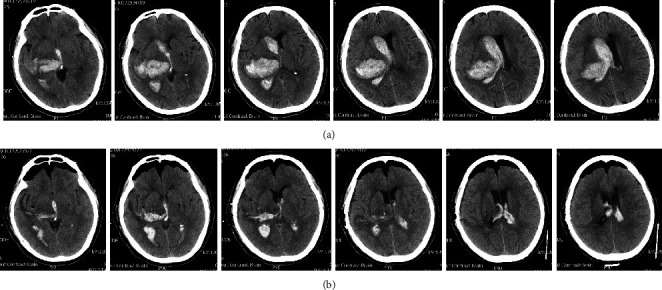
Axial CT scans. (a) Preoperative thalamic hematoma which ruptured into the ventricle. (b) Postoperative residual thalamic hematoma and intraventricular hematoma.

A 71-year-old man was hospitalized due to sudden onset of left hemiparesis. He was brought to the emergency department within an hour. His consciousness level deteriorated soon from an initial GCS score of E3V5M6 to a score of E2V1M5, with preferential gaze to the right side. The CT scan revealed right thalamic hemorrhage with IVH and acute obstructive hydrocephalus. The volume of the hematoma was around 50 mL (Figure 4(a)). The patient was treated with MIS evacuation of ICH and IVH. The postoperative CT scan revealed minimal (around 5 mL) thalamic hematoma at the right side and residual hematoma at ventricles (Figure 4(b)). Bilateral EVDs were kept for three days after the operation, with no ventriculoperitoneal *shunt* implantation or intraventricular injection of anticoagulants. His GCS score was improved to E4V2M6 at one month after the surgery.

## 4. Discussion

According to the timing of surgery, surgeries can be classified into ultraearly (within 6 h after ICH), early (within 6–24 h after ICH), and delayed (4 days after ICH) surgeries [6, 9]. In this retrospective study of seven patients receiving ultrarapid MIS evacuation of thalamic hemorrhage, we observed a good surgical outcome, with no postoperative rebleeding or death noted. In addition, consciousness level and functional outcomes were improved after the surgery. Our data suggested that ultrarapid evacuation of thalamic hemorrhage via MIS may be an optimal surgical approach for selected patients with thalamic hemorrhage.

According to the American Heart Association/American Stroke Association guidelines for the management of spontaneous ICH [10], in patients with IVH and hydrocephalus, ventricular drainage is reasonable, especially for those with decreased level of consciousness. Whether or not removal of thalamic hematoma will improve outcome is still controversial. In recent years, we have performed aggressive evacuation of thalamic hematoma along with IVH removal in early thalamic ICH evacuation. Most of the surgeries were done within 6 hours after the onset of stroke. Since EVD is the most frequently used for thalamic hemorrhage with IVH, we took the EVD group as the matched control in this study, of which the ICH score is similar with the MIS group. Despite the patients receiving MIS evacuation bear higher volume of thalamic hemorrhage, the result showed that patients underwent early MIS evacuation had no rebleeding, lower shunt dependency, no tracheostomy rate, and shorter ICU stay as well as hospital stay. Moreover, better functional outcome was revealed in the MIS group. This result is compatible with the study conducted by Scaggiante et al. [11], which concluded that with a time to evacuation within 72 hours and 24hours, morbidity and mortality improved significantly in the MIS group over the other treatment group. Moreover, our study suggested that with a time to evacuation within 6 hours, MIS evacuation demonstrated efficacy over EVD placement.

It is well known that the local mass effect of hematoma can contribute to elevate intracranial pressure (ICP) and therefore elicit pathological cascades provoking biochemical toxicity. With the favorable outcome observed in this study, we suggested that the early evacuation of ICH can stop the expansion of initial hematoma and prevent associated pathological cascades. Similarly, a previous study has proved that decreasing the clot size to 15 mL or less may ameliorate the functional outcome [12]. Another study also demonstrated that early and minimally invasive gross-total removal of ICH can decrease the ICH-associated secondary injury [13].

However, according to the American Heart Association/American Stroke Association guidelines, timing to perform surgery for ICH remains controversial; furthermore, it was suggested that ultrarapid surgery may escalate the possibility of rebleeding. However, this statement was established on the limited evidence from one study comparing 11 patients undergoing traditional craniotomy within 4 hours of ICH onset with those undergoing traditional craniotomy within 12 hours. The rebleeding rate was 40% in the early surgery (within 4 hours) group, whereas the rebleeding rate was 12% in the late surgery (within 12 hours) group [14]. However, patients' characteristics, ICH severity, surgical method, timing of surgery, and surgeon's experience are all critical prognostic factors. It is inappropriate to conclude that an ultraearly surgery is detrimental to ICH patients. Moreover, unlike our study using the MIS, which has the advantage of minimizing the blood loss, the study applied the traditional craniotomy, which may incur more blood loss and increase the risk of rebleeding. For MIS, the rebleeding problem can be simply solved by applying local hemostatic matrix [7].

In addition to direct injury, ICH can cause secondary brain injury through late-phase inflammatory reactions. Previous studies have proved that degradation of hemin (the oxidized form of heme) can cause cell or brain tissue damage through direct cytotoxic effects [15].The key enzyme of heme catabolism is heme oxygenase-1 (OH-1), which is activated after the onset of ICH. OH-1 catalyzes heme and produces free iron, which is further oxidized into Fe^3+^ and contributes to oxidative stress, brain edema, neuronal death, and blood-brain barrier damage. Several animal studies have shown that OH-1 can be rapidly activated at 6 hours after ICH and reaches a peak at 3 to 7 days [16, 17]. In clinical studies, Liu et al. have discovered a significant increase of OH-1-positive cells, OH-1 protein expression level, and OH-1 RNA transcription level at 6 hours after the onset of ICH. In addition, inflammatory cytokines (e.g., TNF-*α*, IL-1, and IL-10) were also increased significantly at 6 hours after the onset of ICH, which led to the increase of cell apoptosis in brain tissues surrounding the hematoma [18]. These findings may indicate that hematoma evacuation within 6 hours after ICH has the potential to alleviate the cytotoxic cascade caused by heme degradation and further improve the surgical and functional outcomes.

Hematoma volume, initial GCS, and the presence of acute hydrocephalus are generally considered the prognostic factors of ICH evacuation. However, our preliminary result shows low correlation between hematoma volume and 1-year mRS and moderate correlation between initial GCS score and 1-year mRS. This contradicted to previous studies which proved that hematoma volume is the most powerful determinant of outcome [19]. One possible explanation for the differing results may have to do with the fact that we performed MIS instead of traditional craniotomy. Technically, it is difficult to remove the thalamic hemorrhage by traditional craniotomy as it may incur worse prognosis, especially for large hematoma [20, 21]. In contrast, MIS can be done quickly (within 1.5 hours) to prevent secondary injury and cause minimal damage to the brain tissue, which may benefit the functional outcomes.

Since the thalamus is anatomically proximal to the brainstem and ventricle, patients with thalamic hemorrhage have a higher chance of intraventricular extension (which leads to IVH and hydrocephalus) and midbrain compression or destruction, which may lead to the poor prognosis. Likewise, previous studies have reported that thalamic hemorrhage is inherited with poor outcome than other types of ICH [4, 7, 22]. To evacuate thalamic hemorrhage efficiently while preserving functions, our primary goal was to ease the acute hydrocephalus and elevated ICP. In this study, thalamic hemorrhage volume were >30 mL in most cases, which affected not only the thalamus but also proximal structures such as internal capsule, putamen, globus pallidus, and ventricles. We entered the thalamus only after identifying the rupture site during surgery. Such concept of taking IVH evacuation as the priority has been proved to result in better clinical outcomes, including lower shunt-dependent rate, lower pneumonia rate, and higher GOSE score [23].

Theoretically, to remove IVH as the priority may lead to lower clearance rate of thalamic hematoma; however, some of our cases demonstrated a high clearance rate of up to 90% or above. Before the thalamic hematoma hardens, the hematoma would enter the ventricle through the rupture site, where we could use the suction to evacuate the hematoma. It is opposite to the common belief that delayed evacuation is technically simpler due to partial liquefaction of the hematoma. Our concept concurs with a study which suggests that operation should be conducted in an ultrarapid manner within 24 hours after the onset of ICH since ICH usually begins to harden about 24 hours after the onset [24].

In fact, thalamic hemorrhage in most patients of this study expended laterally to critical functional areas, such as internal capsule and lentiform nucleus. Some neuroanatomical structures, such as corticospinal tract that passes through posterior limb of internal capsule, were damaged due to the hemorrhage expansion. Although we performed ultraearly MIS after the onset of ICH, the damage in critical areas had already occurred, which may explain the poor functional outcomes (mRS > 3) observed in some patients. Such findings were also reported and discussed in some studies [25, 26].

This study has some limitations. First, the selection of patients may result in biases. This study excluded patients with poor prognosis, such as patients with a lower GCS score (≤3), with trauma, with coagulopathy, or receiving antiplatelets or anticoagulants, and there were no patients undergoing MIS at more than 6 hours after the onset. Second, this study had a small sample size of seven patients only. With these limitations, the study results should be interpreted with caution. Further large-scale investigation is essential to confirm the findings in our study.

## 5. Conclusions

The timing to perform MIS is an important factor for surgical and functional outcomes. Although the evacuation of thalamic ICH is difficult due to the location, the results in this research indicate that MIS evacuation of thalamic hemorrhage within 6 hours is safe and effective. Moreover, this study shows that it led to improved outcome in selected patients. To our knowledge, this is the first study to focus on the benefit of ultrarapid MIS evacuation for thalamic hemorrhage.

## Figures and Tables

**Table 1:** Demographics, clinical characteristics, and outcomes.

Parameters	MIS (*N* = 7)	EVD (*N* = 7)
Age (years), mean ± SD	66.6 ± 10.5	68.2 ± 12.4
Male, *n* (%)	4 (57.1)	2 (28.6)
ICH volume (mL), mean ± SD	35 ± 8.5	18.0 ± 8.5
ICH score, mean ± SD	3.0 ± 0.76	2.3 ± 0.5
Evacuation rate (%), median	74.9	—
Operation time (min), mean ± SD	116.4 ± 37.7	61.0 ± 18.1
Time to operation (hours)	3.1 ± 0.95	6.0 ± 4.5
Within 6 hr, *n* (%)	7 (100.0)	5 (71.4)
Within 6-24 hr, *n* (%)	0 (0.0)	2 (28.6)
ICU stay	20 ± 8.3	22 ± 7.8
Hospital stay	34.6 ± 13.5	48.7 ± 17.7
Initial GCS, median	8	8
1-month GCS, median	10	11
6-month GCS, median	10	12
1-year GCS, median	10	12
6-month GOSE, median	3	3
6-month mRS, median	5	5
1-year GOSE, median	3	3
1-year mRS, median	4	4
Rebleeding, *n* (%)	0 (0.0)	1 (14.3)
Morbidity, *n* (%)	1 (14.3)	4 (57.1)
VP shunt	1 (14.3)	4 (57.1)
Tracheostomy	0 (0.0)	4 (57.1)
Death, *n* (%)	0 (0.0)	0 (0.0)

Abbreviations: SD: standard deviation; ICH: intracerebral hemorrhage; GCS: Glasgow Coma Scale; GOSE: Glasgow Outcome Scale Extended; mRS: modified Rankin Score.

## Data Availability

The data used to support the findings of this study are available via contacting the corresponding author.
